# Identification of *OCTN2* variants and their association with phenotypes of Crohn’s disease in a Korean population

**DOI:** 10.1038/srep22887

**Published:** 2016-03-11

**Authors:** Hyo Jin Park, Eun Suk Jung, Kyoung Ae Kong, Eun-Mi Park, Jae Hee Cheon, Ji Ha Choi

**Affiliations:** 1 Department of Pharmacology, Tissue Injury Defense Research Center, School of Medicine, Ewha Womans University, Seoul, 07985, Korea; 2 Department of Pharmacology, Brain Korea 21 PLUS Project for Medical Sciences, Severance Biomedical Science Institute, Yonsei University College of Medicine, Seoul, 03722, Korea; 3 Department of Internal Medicine and Institute of Gastroenterology, Yonsei University College of Medicine, Seoul, 03722, Korea; 4 Clinical Trial Center, Ewha Womans University Medical Center, Seoul, 07985, Korea

## Abstract

Crohn’s disease (CD) is a chronic inflammatory bowel disease and a genetic variant in the *OCTN2*, g.-207G > C is significantly associated with CD susceptibility. This study was aimed to identify novel *OCTN2* functional promoter variants and their roles in transcriptional regulation using various *in vitro* assays. In addition, we investigated the association between *OCTN2* genotypes and CD through genetic analysis using DNA samples from 193 patients with CD and 281 healthy controls. Among the three major promoter haplotypes of *OCTN2* identified, one haplotype, H3, showed a significant decrease in promoter activity: two polymorphisms in H3 were associated with a significant reduction in promoter activity. In particular, we found that the reduced transcriptional activity of those two polymorphisms results from a reduction in the binding affinity of the activators, NF-E2 and YY1, to the *OCTN2* promoter. The functional haplotype of the *OCTN2* promoter was associated with clinical course of CD such as the disease behavior and need for surgery. However, genetic variants or haplotypes of *OCTN2* did not affect the susceptibility to CD. Our results suggest that a common promoter haplotype of *OCTN2* regulates the transcriptional rate of *OCTN2* and influences the clinical course of CD.

Crohn’s disease (CD), which is categorized as an inflammatory bowel disease (IBD), is an immune-mediated disorder caused by a combination of genetic and environmental factors^1^. In addition to smoking, which is the most common environmental risk factor for CD^2^, several environmental factors, such as hygiene, breastfeeding, antibiotic use, and the westernized diet, are known to promote the risk of CD^3^,^4^,^5^,^6^. Such environmental risk factors are considered important; however, genetic backgrounds that predispose to CD should also be considered similarly important as demonstrated in twin and familial studies and in ethnic-specific studies^7^,^8^. In particular, recent studies of immigrants investigating the role of environmental risk factors in CD susceptibility revealed that certain ethnic groups, especially those from Asia, show low incidence of CD despite being exposed to similar environments as other groups showing greater incidence of the disease^9^. These distinct, population-specific patterns in CD suggest that the collection of sufficient information from ethnic-specific genetic studies may be highly useful for understanding the ethnic specific genetic pathogenesis of CD^10^,^11^.

The advances in genetic approaches, such as genome-wide association studies, have resulted in the identification of 140 genes and loci related to CD and IBD in the human genome^12^. Previous genetic studies have shown CD to be associated with a broad range of genes such as *NOD2, ATG16L1, IRGM, IBD5*, and *TNFSF15*^13^,^14^,^15^,^16^,^17^. Of these, NOD2, an intracellular pattern recognition receptor and inducer of autophagy, and autophagy-related proteins, such as ATG16L1 and IRGM, are known to be involved in the pathogenesis of CD; suggesting that autophagy may play an important role in maintaining host-microbe interactions in intestinal epithelium^18^,^19^. Certain *TNFSF15* polymorphisms are not only associated with susceptibility of CD in several populations^20^,^21^, but also with clinical prognosis, indicating that the genetic information of the patient is an independent predictive factor^22^. In accordance with these studies, it was proposed that abnormal immune reactions and chronic inflammation might occur in the intestinal epithelium when various environmental factors disrupt the host-microbe homeostasis in genetically susceptible individuals, resulting in the development and progression of CD^1^. However, the pathogenesis of CD and the roles of the proteins described above remain poorly understood.

The organic cation/carnitine transporter 2 (OCTN2, encoded by Solute Carrier Family 22 Member 5, *SLC22A5*), located on chromosome 5q31 in the *IBD5* region, is a polyspecific membrane transporter^23^ expressed ubiquitously on cells, such as those of the kidney, skeletal muscle, placenta, heart, pancreas, liver, lung, intestine, and brain^24^. Recent studies demonstrate that the OCTN2 expression levels in inflamed regions of intestinal epithelium were higher than in non-inflamed areas^25^. OCTN2 mediates bidirectional transport, depending on the substrate e.g., carnitine, the cofactor involved in β-oxidation of fatty acid in mitochondria, is transported from extracellular to intracellular space in a sodium-dependent manner, whereas tetraethylammonium is transported in the opposite direction in a proton-dependent manner^26^. Moreover, the ability of OCTN2 to transport various xenobiotics, such as verapamil, cephaloridine, and oxaliplatin, may make this protein potentially useful in the fields of drug pharmacokinetics and pharmacodynamics^27^,^28^,^29^. In addition, several *OCTN2* variants have been demonstrated to show an association with human diseases such as primary systemic carnitine deficiency (CDSP), asthma, and CD^17^,^30^,^31^. For instance, multiple *OCTN2* variants were found to be associated with CDSP, which symptoms include progressive skeletal myopathy and cardiomyopathy due to insufficient uptake of carnitine, confirming *OCTN2* to be a major causative gene of this disease^32^.

In spite of the clinical importance of the OCTN2 transporter, few functional single nucleotide polymorphisms (SNPs) of *OCTN2* have been identified and characterized e.g., F17L, a nonsynonymous polymorphism of *OCTN2*, is known to encode a partially functional form of the transporter that is deficient in plasma membrane trafficking, which directly affects its uptake^33^. In particular, to date, there has been little data characterizing the functional promoter variants of *OCTN*2. In a previous study, Peltekova *et al.* reported the association between the susceptibility to CD and TC haplotype of *OCTN*s, i.e. *OCTN1* L503F and *OCTN2* g.-207G > C^17^. This finding was subsequently validated by several association studies in diverse populations^34^,^35^,^36^,^37^,^38^,^39^. However, it is known that these two *OCTN* polymorphisms are absent in Asian populations^37^,^38^,^39^.

In the present study, we aimed to identify and characterize the SNPs in the promoter region of *OCTN2* and investigate the mechanisms involved in transcriptional regulation of this gene. Additionally, we investigated the association between *OCTN2* functional promoter variants and CD: their relevance to its clinical course and susceptibility of CD in the Korean population.

## Results

### Identification of genetic variants and haplotypes of the *OCTN2* promoter region

In order to identify genetic variants of the *OCTN2* promoter region in the Korean population, a 2,166 base pair (bp) sequence was directly sequenced in 48 Korean genomic DNA samples. Ten *OCTN2* promoter variations were found with minor allele frequency ranging from 0.021 to 0.427, and one of these (g.-299G > C) was found to be novel (Table 1). Names of all variants in this study conformed to the Human Genome Variation Society nomenclature. Furthermore, we compared the frequencies of those variants in other Asian populations -Han Chinese (CHB) and Japanese (JPT)- using genotype data from the 1000 Genomes Project (phase 3) (The genotype data for specific SNPs was obtained from the 1000 Genomes Project using the 1000 Genomes Browser, version 3.5, provided by National Center for Biotechnology Information (NCBI), https://www.ncbi.nlm.nih.gov/variation/tools/1000genomes/) and observed that both populations have frequencies comparable to that of the Koreans. In particular, there was a high similarity among the frequencies in the Chinese and Korean populations (Supplementary Table S1). Previous genetic studies of the *OCTN2* gene focused on the association between the common *OCTN2* polymorphism g.-207G > C and CD^17^,^40^,^41^. However, g.-207G > C was not observed in our study. To our knowledge, except for the study on the variant g.-207G > C, the functions of genetic variants of the *OCTN2* promoter have not been investigated to date, although Tahara *et al.* previously reported the functional effect of the proximal promoter (−253 to +89 bp from the translational start site) of *OCTN2*^42^. Three major (with frequency above 5%) haplotypes (H1 to H3) were found in our study population after haplotype assembly. The haplotypes’ frequencies are listed in Table 2. Among these, the H1 haplotype with the highest frequency (40.6%) was regarded as a reference haplotype in the present study, based on NM_003060.3 from the database of SNPs of the NCBI. In addition, haplotype assembly was conducted using genotype data obtained from three ethnic groups: Americans of African ancestry (ASW), Utah residents with European ancestry (CEU), and CHB (https://www.ncbi.nlm.nih.gov/variation/tools/1000genomes/). We found that three ethnic groups possessed *OCTN2* promoter haplotypes identical to the three major haplotypes mentioned above, with the exception of ASW that has one additional H4 haplotype, the frequency of which is also ≥5%. However, we observed that the frequencies of *OCTN2* promoter haplotypes in Koreans were more similar to those of CHB, in comparison to ASW or CEU populations (Supplementary Table S2 and Figure S1).

### Functional effects of haplotypes and variants on *OCTN2* promoter activity

**Figure 1:**
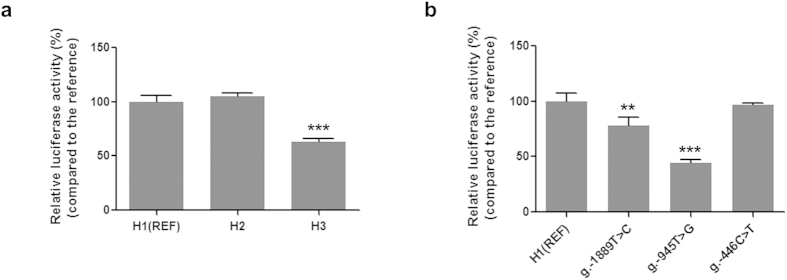
Luciferase activities of the haplotypes or genetic variants of *OCTN2* promoter region. The promoter activities were measured 48 hours after transient transfection of the reporter plasmids containing the major *OCTN2* haplotypes (**a**) or genetic variants (**b**) into HCT-116 cells. Then, the luciferase activity of each construct was compared with that of the reference (REF, H1). The data (mean ± SD) were obtained from triplicate wells. ^**^P < 0.01, ^***^P < 0.001 vs. reference promoter activity.

In order to characterize the *OCTN2* promoter variants, reporter plasmids containing either the reference or other haplotype sequences of *OCTN2* promoter region were constructed. These reporter plasmids were transiently transfected into human colorectal carcinoma (HCT-116) cells and their promoter activities were measured by luciferase assay. In the previous study, *OCTN2* promoter activities of four cell lines, ACHN, HEK-293T, LLC-PK1, and HCT-116, were examined. Among them, the OCTN2 reference-HCT-116 cells were found to show the highest promoter activity^42^. Therefore, HCT-116 cells were used to perform reporter assays in this study. We found that one of the major promoter haplotypes, H3, showed a significantly decreased promoter activity (*P* < 0.001) (by 37.0% compared to that of the reference, H1), whereas the promoter activity of another haplotype, H2, was comparable with that of the reference (Fig. 1a). Then, in order to identify variants involved in reducing H3 promoter activity, vectors containing each variant present in H3 were constructed and their activities were measured. Two polymorphisms (g.-1889T > C and g.-945T > G) in H3 showed significantly reduced promoter activities (decreased by 21.9% and 55.4%, respectively) (Fig. 1b). However, there was no difference in promoter activity between the g.-446C > T and the reference. These findings suggest that H3 shows reduced promoter activity with respect to reference due to the effects of two polymorphisms: g.-1889T > C and g.-945T > G.

### Investigation of potential transcription factors binding to the *OCTN2* promoter

**Figure 2:**
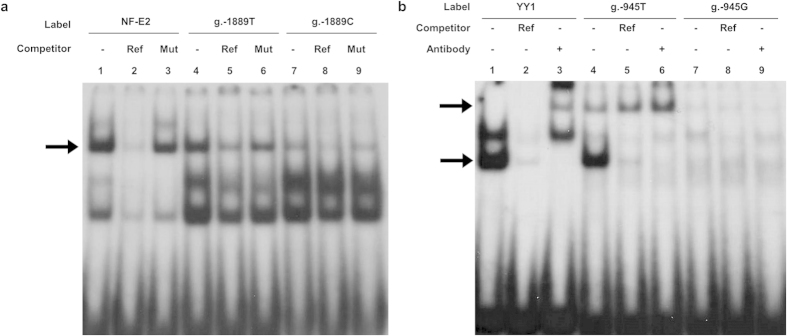
Identification of interaction between transcription factors and genetic variants on *OCTN2* promoter through EMSA. (**a**) ^32^P-labeled oligonucleotides (lanes 1–3, NF-E2 consensus; lanes 4–6, g.-1889T reference; lanes 7–9, g.-1889C variant) were incubated with 10 μg of nuclear protein extracts obtained from HCT-116. Competition assay was performed using 100-fold molar excess of unlabeled NF-E2 consensus oligonucleotides (lanes 2, 5, and 8) or unlabeled mutant NF-E2 oligonucleotides (lanes 3, 6, and 9). The arrow indicates the band of the DNA-protein complex. (**b**) ^32^P-labeled oligonucleotides (lanes 1–3, YY1 consensus; lanes 4–6, g.-945T reference; lanes 7–9, g.-945G variant) were incubated with 10 μg of nuclear protein extracts. Competition assay was performed using 100-fold (lanes 2, 5, and 8) molar excess of unlabeled YY1 consensus oligonucleotides. Supershift experiments were performed with 2 μg of the antibody against YY1 (lanes 3, 6, and 9). The lower arrow indicates the position of the DNA-protein complex and upper arrow indicates supershift band that formed the complex including labeled probe, YY1 protein, and anti-YY1 antibody (lanes 3 and 6). None of the shift or supershift bands with g.-945G variant were shown in this experiment due to weak binding affinity with the sequence.

In order to further investigate the mechanism underlying the transcriptional regulation of the *OCTN2* promoter, prediction of potential transcription factor binding sites for the two variants, g.-1889T > C and g.-945T > G was performed by *in silico* analysis using TFSearch (version 3.1, Real-World Company Partnership, Tokyo, Japan), ConSite (http://consite.genereg.net/cgi-bin/consite), and MatInspector (Genomatrix Software GmbH, Munich, Germany). Two transcription factors, nuclear factor erythroid 2 (NF-E2) and Yin Yang 1 (YY1), were predicted to bind to the *OCTN2* promoter and show a large difference in binding affinity between reference and variant sequences. In order to confirm this prediction, electrophoretic mobility shift assays (EMSAs) were performed using nuclear extracts from HCT-116 cells and ^32^P-labeled oligonucleotides. We observed that NF-E2 bound to the reference g.-1889T 1.53-fold more strongly than the variant g.-1889C (lanes 4 and 7, Fig. 2a). The marked bands were ascertained to correspond to the NF-E2-oligonucleotide complex by competition assay, in that a 100-fold concentration of unlabeled consensus oligonucleotides competed with the labeled consensus (lane 2, Fig. 2a), *OCTN2* reference (lane 5), or variant (lane 8) probes. However, these binding complexes were unable to compete with a 100-fold concentration of unlabeled oligonucleotides containing mutated core sequences of each transcriptional factor (lanes 3, 6, and 9, Fig. 2a). For the g.-945T > G polymorphism, the TFSearch and ConSite programs predicted that YY1 preferentially interacts with the reference sequence (g.-945T) over the variant (g.-945G). The result from EMSA was consistent with this prediction, in that the variant g.-945G was poorly represented in the band corresponding to YY1-probe complex (lanes 1, 4, and 7, Fig. 2b). In addition, the supershift in the presence of antibodies against YY1 confirmed that YY1 was present in the complex (lanes 3 and 6, Fig. 2b).

### NF-E2 and YY1 activate transcription from the *OCTN2* promoter

**Figure 3:**
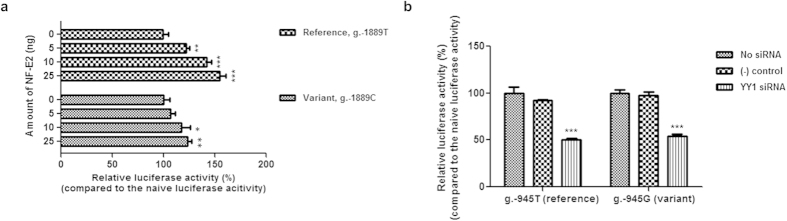
Effect of NF-E2 and YY1 on *OCTN2* promoter activity. (**a**) Promoter activities were measured 48 hours after co-transfection of reference or variant plasmids and various amounts (0 to 25 ng) of *NF-E2* into HCT-116 cells. The reporter activity of each construct was compared with naïve promoter activity without NF-E2. (**b**) Twenty-four hours after reverse transfection using siRNA for YY1, reference or variant luciferase constructs of *OCTN2* promoter were co-transfected into HCT-116 cells. Forty-eight hours after the transfection, luciferase activities were measured. The reporter activity of each construct was compared with naïve promoter activity without siRNA. The data shown represent mean ± SD from triplicate wells in a representative experiment. ^*^P < 0.05, ^**^P < 0.01, ^***^P < 0.001 vs. naive promoter activity.

In order to examine the effect of NF-E2 on *OCTN2* transcription, luciferase assays were conducted following overexpression of NF-E2. The results showed NF-E2-activated *OCTN2* promoter activity in a dose-dependent manner. In particular, the effect of NF-E2 on the promoter activity of *OCTN2* with the reference sequence was larger than that of *OCTN2* containing the variant sequence. This finding was consistent with the EMSA results. Specially, when 25 ng of *NF-E2* was co-transfected with reporter plasmids, the promoter activity of *OCTN2* containing g.-1889T was increased by 55.1%, while the promoter activity of *OCTN2* containing g.-1889C was increased by 24.0% (Fig. 3a). The effect of YY1 on the *OCTN2* promoter activity was examined by using small interfering RNA (siRNA) to knockdown YY1. After 24 hours of siRNA transfection to HCT-116, the reference (g.-945T) or variant (g.-945G) reporter constructs were transfected into the cells. Then, luciferase activities were determined after 48 hours. As shown in Fig. 3b, there was a significant reduction in luciferase activities of the *OCTN2* reference and variant in the presence of YY1 siRNA compared to the luciferase activities with the negative control siRNA. These data suggest that NF-E2 and YY1 act as activators on the *OCTN2* transcription.

### Effect of *OCTN2* functional promoter variants on CD susceptibility

We examined whether the functional *OCTN2* promoter variants affected the susceptibility to CD in Koreans. Genetic analysis of *OCTN2* promoter variants was performed using DNA samples from 193 patients with CD and 281 healthy controls. Then, we compared the frequency of each variant or haplotype between the two groups and found that the frequencies of two functional variants of the *OCTN2* promoter were similar between the two groups (Supplementary Table S3). In addition, there was no significant difference in the frequencies of the three *OCTN2* promoter haplotypes, involving the two functional variants, between the two groups (Table 3).

### Effect of *OCTN2* functional promoter variants on the clinical phenotypes of CD

**Figure 4:**
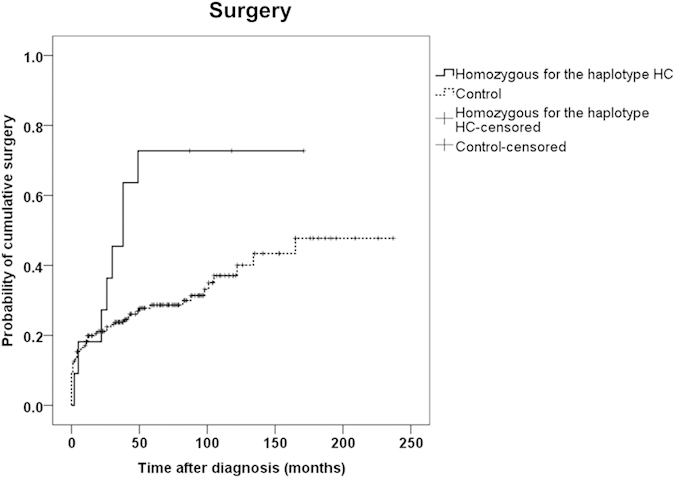
Probability of cumulative surgery curve in Korean CD patients during follow-up. The univariate Kaplan-Meier log-rank test was performed between homozygous for *OCTN2* haplotype HC (solid line) and control (dashed line). log-rank *P* = 0.015.

Previous studies reported that genetic variants of several genes such as *IL23R*, *PRDM1*, *NOD2*, and *TNFSF15* were significantly associated with clinical phenotypes of CD^43^. Here, we investigated the association between functional variants of *OCTN2* promoter and the clinical course of CD in Koreans. In particular, we hypothesized that the *OCTN2* functional variants that showed decreased promoter activity could be associated with a more severe clinical course of CD, as well-known *OCTN1* L503F and an *OCTN2* g.-207G > C, both showed a reduction in transport or promoter activity, respectively, in a previous study^17^. In addition, previous studies reported that OCTNs play protective roles against toxic stresses in intestinal epithelial cells^25^,^44^. In our study population, haplotype HC (g.-1889C and g.-945G) in Table 3 was the *OCTN2* haplotype that consists of only variants showing the decreased promoter activity. In order to evaluate the effect of this functional *OCTN2* HC haplotype on clinical phenotypes of CD, we divided 193 patients into two groups: a control group (n = 182) and a variant group (n = 11). The variant group consisted of subjects homozygous for the haplotype HC, and the control group consisted of those that were not members of the variant group. First, we compared the patients’ characteristics according to the *OCTN2* haplotype and observed that there was no significant difference in sex, age of onset, body mass index, and disease location between the two groups (Table 4). In our study population, the average age of the patients was 25 years at the time of diagnosis with CD, and the proportion of men in both groups was higher than that of women. Table 5 shows the clinical course of patients according to the *OCTN2* haplotype. The proportion of patients who underwent surgery was higher in the variant than in the control group [odds ratio (OR) = 7.542, *P* = 0.004, adjusted *P* = 0.016 after Benjamini-Hochberg correction^45^]. In addition, we found that the *OCTN2* haplotype HC was associated with penetrating disease behavior (OR = 4.239, *P* = 0.042, adjusted *P* = 0.084 after Benjamini-Hochberg correction); 45.5% of the patients in the variant group experienced penetrating behavior, compared to only 19.2% in the control group. In our study population, five specialists in internal medicine diagnosed the patients as having CD. Among them, two doctors diagnosed almost all the patients (92% of total). We divided patients into three groups and compared the proportion of patients who underwent surgery or got azathioprine or anti-TNF according to the doctors; the first group consisted of the patients diagnosed by one of the two doctors who diagnosed most of the patients, the second group consisted of the patients diagnosed by the other doctor, and the third group consisted of the patients who did not belong to the first two groups. As a result, we observed that there was no significant difference between the groups (*P* = 0.864 and 0.351, respectively). Finally, we investigated that *OCTN2* haplotype and clinical factors associated with the time to development of these outcomes. Results of time to clinical events of the univariate Kaplan-Meier log-rank test analysis are shown in Supplementary Table S4. Of these, time to surgery was significantly associated with *OCTN2* haplotype HC (log-rank *P* = 0.015, Fig. 4).

## Discussion

To date, several association studies between CD and *OCTN* gene variations have been conducted; however, the precise mechanisms underlying the regulation of these genes remain obscure. Peltekova *et al.* suggested that L503F in *OCTN1* might result in the change of transporter functions and g.-207G > C in *OCTN2* may destroy the heat-shock transcription factor binding element, decreasing the expression of the gene^17^. In the present study, we found a common *OCTN2* promoter haplotype showing decreased promoter activity, and identified two transcription factors, NF-E2 and YY1, involved in this regulation. NF-E2, which belongs to the basic-leucine zipper family and is known to function as a transcription regulator in globin synthesis, megakaryocyte maturation, and platelet production, is additionally associated with atherosclerosis, immune responses, and inflammation^46^,^47^. The consensus sequence of NF-E2 is TGCTGAS(c/g)TCAY(c/t) and the sequence of its binding site on the *OCTN2* promoter is TCTTTACTCAA^48^. These sequences are similar, and matched nucleotides with consensus are underlined. The occurrence of the variant, g.-1889T > C in this region changes the sequence to TCTTTACCCAA, which is expected to lower the binding affinity of NF-E2. This prediction was confirmed by EMSA. Additionally, we observed that NF-E2 activates transcription of *OCTN2*. The other transcription factor identified to play a role in the regulation of *OCTN2* activity was YY1. YY1 is a well-known, ubiquitously expressed transcription factor that plays various roles in different genes and cellular environments e.g., YY1 was found to positively regulate the expression of a wide range of genes such as BRCA1, TGF-β, and BACE1, with broad phenotypical effects such as tumor formation and progression of Alzheimer’s disease^49^,^50^,^51^. In the present study, we found that YY1 functions as an activator of *OCTN2* transcription.

Although the pathogenesis of CD is not fully understood, several previous reports have implied that OCTN2 is involved in the pathogenesis of CD. OCTN2 was initially widely recognized as being expressed in intestinal epithelium where it plays a role in transporting various substrates^26^. This finding suggests that the pathogenesis of CD may be linked to impairment of the integrity of membrane barrier of intestinal epithelial cells. OCTN2 was also observed to play a role in the transport of substrates, which are known to be involved in detoxification or other protective mechanisms e.g., intestinal OCTN2 mediated the uptake of competence and sporulation factor that prevents intracellular oxidant-related damage and loss of barrier function^52^. Furthermore, if OCTN2 function related to the transport of serotonin is disrupted, the balance between serotonin and dopamine across the epithelium is destroyed, increasing the risk of serotonin toxicity in cells^53^. Finally, it may be assumed that when several toxic stresses are present in the intestinal epithelium, the expression or activity of OCTN2 is increased via a feedback mechanism. Fujiya *et al.* revealed that the expression of OCTN2 was increased in inflamed areas in order to compensate for cellular damage^25^. Collectively, these findings suggest that OCTN2 plays a protective role against toxic stresses in intestinal epithelial cells, as a result of which individuals with genetic variants of *OCTN*2 are susceptible to intestinal inflammation, with a worse prognosis.

In the present study, we observed that the *OCTN2* haplotype HC showed reduced promoter activity, and which was associated with higher proportion of patients undergoing surgery and higher penetrating behavior. In other words, we found that patients with functional *OCTN2* haplotype showed a more severe clinical course as compared with the control. Several groups have reported similar effects of the *OCTNs* TC haplotype on the clinical course of CD in diverse populations^40^,^41^,^54^,^55^. e.g., Noble *et al.* found that the TC haplotype of *OCTNs* was significantly associated with stricturing and penetrating disease, and higher frequency of having surgery, as CD patients with this haplotype tended to show severe progression of disease^40^.

Present study has several limitations. First, all participants in this study were of East-Asian descent. Second, we did not collect information on the environmental factors influencing CD, as smoking behavior of all the participants, because the genotype-phenotype analysis has been performed retrospectively. However, we compared the number of smokers between 9 patients from the variant group and 122 patients from the control group, according to the *OCTN2* haplotype, and observed no significant differences in the smoking history between the two groups (33% in the variant vs. 36% in the control groups, *P* = 1.000). Finally, the sample size was not large enough to reach sufficient statistical power. Therefore, although a high strength of association (OR = 4.239) between the *OCTN2* haplotype and penetrating behavior was observed, the effect of this haplotype on penetrating behavior was not statistically significant when the Benjamini-Hochberg correction for multiple comparisons was applied to the analysis. On the contrary, the need of surgery was significantly associated with the haplotype even after the correction. Therefore, in our opinion, these findings indicate that this haplotype is associated with a severe course of CD. In particular, the present study is strengthened by the fact that we identified for the first time possible disease-associated variants from the population data and investigated the effect of these variants on the gene function at the molecular level. Next, we performed a genotype-phenotype association analysis to validate our hypothesis based on data from *in vitro* assays.

In conclusion, we identified functional variants and haplotypes in promoter region of *OCTN2*, and found that the association between the functional promoter variants in *OCTN2* and susceptibility to CD was not statistically significant. However, we observed that the functional *OCTN2* promoter haplotype significantly affected the clinical course of CD. In particular, this haplotype was strongly associated with the need for surgery in CD patients. To our knowledge, this is the first study examining the relationship between promoter haplotype in *OCTN2* and susceptibility or clinical phenotypes of CD in Koreans. Further studies with various ethnic populations would be necessary to confirm this haplotype as a useful prognosis predictor in CD. In addition, it may be useful to investigate whether the functional haplotype of *OCTN2* promoter is associated with susceptibility to, or clinical features of, other diseases related to chronic inflammation or the immune response.

## Methods

### Genetic analysis of *OCTN2* promoter

This study was reviewed and approved by the Institutional Review Board of the Ewha Medical Center, Seoul, Korea. All experiments were performed in accordance with relevant guidelines and regulations of Institutional Review Board of the Ewha Medical Center. In order to identify the genetic variants of the *OCTN2* promoter, a 2,166 bp PCR fragment (−2,354 to −189 bp from the translation start site) was amplified and directly sequenced using DNA samples, obtained from the DNA bank of the Korea Pharmacogenomics Research Network at Seoul National University, Seoul, Korea, from 48 unrelated healthy Korean individuals. Haplotype assembly and calculation of the frequency of each haplotype were performed using the Haploview program (version 4.3, Broad Institute, Cambridge, MA, USA). The genotype data of *OCTN2* promoter variants in other ethnic groups was obtained from the 1000 Genomes Project (phase 3) (https://www.ncbi.nlm.nih.gov/variation/tools/1000genomes/): data from 103 CHB and 104 JPT individuals was used to compare the frequencies of *OCTN2* promoter variants between Asian populations. In addition, LD structures of the *OCTN2* variants were illustrated using data from 61 ASW, 99 CEU, and 103 CHB individuals. Nucleotide location numbers were assigned from the translational start site based on the *OCTN2* mRNA sequence (GenBank accession number; NM_003060.3).

### Cell culture

HCT-116 cells were obtained from the Korean Cell Line Bank (KCLB, Seoul, Korea). Cells were grown in RPMI 1640 medium supplemented with 10% inactivated fetal bovine serum, 100 U/ml penicillin, and 100 μg/ml streptomycin, and maintained in incubator at 37 °C in 5% CO_2_ (WELGENE Inc., Seoul, Korea).

### Construction of reporter plasmids with the *OCNT2* promoter sequence and its variants

In order to generate a luciferase reporter plasmid containing the promoter region of *OCTN2* reference sequence, a 2,618 bp of the human *OCTN2* gene, extending from −2,490 to +128 relative to the translational start site, was amplified from genomic DNA samples from the individual with a reference sequence. Then, the amplified product was inserted between the restriction sites for NheI and KpnI (Enzynomics, Daejeon, Korea) of the pGL4.11 [*luc2P*] vector (Promega Corporation, Madison, WI, USA). The mutant pGL4.11-*OCTN2* vectors containing genetic variants of the promoter region were generated using QuikChange^®^ II Site-Directed Mutagenesis Kit (Agilent Technologies, Santa Clara, CA, USA) following the manufacturer’s instructions, with primers whose sequences are shown in Supplementary Table S5. The sequences of all constructs were confirmed by direct sequencing.

### Measurement of the promoter activity of the variants

The promoter activity of the variants was measured as described in the previous study^56^. Briefly, the HCT-116 cells with 60 ~ 80% confluence were transfected with the reporter plasmids using Lipofectamine LTX and Plus reagents (Life Technologies, Carlsbad, CA, USA). Forty-eight hours after transfection, a Dual-luciferase^®^ reporter assay system and a Glomax 96-well plate luminometer (Promega, Fitchburg, WI, USA) were used to analyze the activity of the promoter variants. Relative luciferase activity was defined as the ratio of activities of firefly and Renilla luciferase.

### EMSA

EMSAs were performed as described previously^57^. In brief 32, P-labeled oligonucleotides (1 × 10^5^ counts/min) were incubated with 10–15 μg of nuclear extract from HCT-116 cells for 30 minutes at room temperature. The oligonucleotides used in the EMSAs are shown in Supplementary Table S5. For the competition assay, the unlabeled NF-E2 and YY1 consensus or mutant oligonucleotides were added in 100-fold molar excess prior to the binding reaction. The samples were loaded on a 6% non-denaturing polyacrylamide gel and electrophoresed for 70 minutes at 80 V. For signal detection, the dried gel was exposed to CP-BU film (Agfa, Mortsel, Belgium) for 16 hours at −80 °C. The intensity of each band was quantified using ImageJ software (National Institutes of Health, Bethesda, MD, USA).

### Co-expression or knockdown of the transcription factors

In order to investigate the effect of NF-E2 on *OCTN2* promoter activity, the *OCTN2* reporter vectors were co-transfected with various amounts (5–25 ng) of the *NF-E2*-pcDNA3.1 vector into HCT-116 cells. *NF-E2* cDNA (OriGene Technologies Inc., Rockville, MD, USA) was subcloned into the pcDNA3.1(+) vector (Life Technologies). The primers used for construction of *NF-E2*-pcDNA3.1 are shown in Supplementary Table S5. In order to achieve down-regulation of YY1 in HCT-116 cells, we performed reverse transfection using siRNA for YY1 (HSS187726) and Lipofectamine RNAiMAX according to the manufacturer’s protocol (Life Technologies). Stealth^TM^ RNAi Negative Control with high GC Duplex (Life Technologies) was used as a negative control.

### Association study

In order to investigate the potential effects of the functional variants of *OCTN2* promoters on the susceptibility or clinical phenotypes of CD, we genotyped two *OCTN2* variants using DNA samples from unrelated patients with CD (Korean, n = 193) and healthy controls (Korean, n = 281). The present study was approved by the Institutional Review Board of Severance Hospital in the Yonsei University Health System, Seoul, Korea. All analyses were performed in accordance with relevant guidelines and regulations of Institutional Review Board of Severance Hospital in the Yonsei University Health System. The patients were diagnosed with CD, and had a follow-up period of at least 24 months with specialists in internal medicine. Written informed consent for participation was obtained from all participants or their guardians prior to enrollment. Clinical data were collected by reviewing the medical records.

### Statistical analysis

All results were presented as means ± standard deviation. Statistical analyses were performed using GraphPad Prism 5.0 (GraphPad Software, Inc., La Jolla, CA, USA) and PASW software v.18.0 and v.21.0 for Windows (SPSS Inc., Chicago, IL, USA). A statistically significant result was defined for *P* < 0.05. *P* values for the luciferase assay were calculated using one-way analysis of variance followed by Dunnett’s two-tailed test. A paired t-test was performed to compare the effects of NF-E2 or YY1 on the luciferase activity of *OCTN2* promoter containing the reference or variant sequences. In order to compare the frequencies of genetic variations or haplotypes between patients and control groups, χ^2^-test was used. The characteristics between patients’ groups according to the *OCTN2* haplotype were analyzed using the χ^2^-test and t-test. The association between the *OCTN2* haplotype and the clinical course of CD was evaluated using binary logistic regression analysis (for azathioprine or anti-TNF agent use and experience of surgery) and multinomial logistic regression analysis (for disease behavior), with adjustment for sex, age of onset, and BMI. In addition, time to clinical events (surgery, disease behavior, and azathioprine or anti-TNF agent use) within the patients was evaluated using the Kaplan-Meier estimate. Finally, the log-rank test was performed to analyze the association between the *OCTN2* haplotype and the cumulative incidence data.

## Additional Information

**How to cite this article**: Park, H. J. *et al.* Identification of *OCTN2* variants and their association with phenotypes of Crohn’s disease in a Korean population. *Sci. Rep.* **6**, 22887; doi: 10.1038/srep22887 (2016).

## Supplementary Material

Supplementary Information

## Figures and Tables

**Table 1:** Frequencies of *OCTN2* genetic variations in promoter region.

rs Number	Variant	Minorallele	Frequency	rsNumber	Variant	Minorallele	Frequency
rs2631372	g.-2087G > C	C	0.313	rs4646298	g.-446C > T	T	0.260
rs2631371	g.-1974G > A	A	0.313	rs2631369	g.-399G > C	C	0.313
rs2631370	g.-1889T > C	T	0.427	rs2631368	g.-368T > G	G	0.313
rs186829555	g.-1679C > G	G	0.021	–	g.-299G > C	C	0.021
rs34786243	g.-945T > G	G	0.255	rs4646300	g.-234C > G	G	0.032

Data was obtained from DNA samples from 48 unrelated Korean individuals.

The position of the variant is based upon the translational start site.

**Table 2:** Frequencies of common *OCTN2* haplotypes in the promoter region.

ID	g.-2087G > C	g.-1974G > A	g.-1889T > C	g.-1679C > G	g.-945T > G	g.-446C > T	g.-399G > C	g.-368T > G	g.-299G > C	g.-234C > G	Frequency
H1	G	G	T	C	T	C	G	T	G	C	0.406
H2	**C**	**A**	**C**	C	T	C	**C**	**G**	G	C	0.280
H3	G	G	**C**	C	**G**	**T**	G	T	G	C	0.240

The SNPs were marked in bold-faced letters.

The minor alleles were marked in letters with underlines.

**Table 3:** Frequencies of functional *OCTN2* haplotypes in patients and control groups.

ID	g.-1889T > C	g.-945T > G	Patient, n (%)	Control, n (%)	OR^a^ (95% CI)	*P* value
HA	T	T	146 (37.8)	209 (37.2)	1.027 (0.786–1.343)	0.843
HB	**C**	T	147 (38.1)	211 (37.5)	1.023 (0.783–1.336)	0.867
HC	**C**	**G**	93 (24.1)	142 (25.3)	0.939 (0.695–1.269)	0.681

^a^OR of patients group for haplotype HA relative to the control group.

CI, confidential interval.

**Table 4:** Characteristics of the patients according to the *OCTN2* haplotype.

Parameter	Haplotype HChomozygosity (%)	Control (%)	Haplotype HChomozygosity(mean ± SD)	Control(mean ± SD)	*P*value
Total number	11	182			
Sex, male	10 (90.9)	130 (71.4)			0.295
Age of onset (years)			24.73 ± 6.87	24.89 ± 9.85	0.957
<16	0 (0.0)	17 (9.3)			
16≤ <40	11 (100.0)	153 (84.1)			0.808
≥40	0 (0.0)	12 (6.6)			
BMI			19.77 ± 2.91	19.92 ± 3.07	0.880
Location					
Ileal involvement	1 (9.1)	27 (14.8)			1.000
Jejunal involvement	4 (36.4)	24 (13.3)			0.058
Colorectal involvement	4 (36.4)	38 (21.0)			0.260
Perianal fistula	6 (54.5)	89 (48.9)			0.765

BMI, body mass index.

**Table 5:** Clinical course of the patients according to the *OCTN2* haplotype.

Parameter	Haplotype HChomozygosity (%)	Control (%)	OR (95% CI)	*P* value^a^
Total number	11	182		
Behavior				
Inflammatory	4 (36.4)	105 (57.7)		
Stricturing	2 (18.2)	42 (23.1)	1.294^b^ (0.226–7.423)	0.772
Penetrating	5 (45.5)	35 (19.2)	4.239^b^ (1.052–17.076)	0.042^c^
Azathioprine or anti-TNF use	9 (81.8)	135 (74.2)	1.608 (0.324–7.968)	0.561
Surgery	8 (72.7)	56 (30.8)	7.542 (1.885–30.172)	0.004^d^

^a^After adjustment for other covariates (i.e. sex, age of onset, and BMI).

^b^OR of multinomial logistic regression model with the inflammatory type as a reference category of outcome variable.

^c^Adjusted *P* = 0.084 after Benjamini-Hochberg correction.

^d^Significant after Benjamini-Hochberg correction (adjusted *P* = 0.016).
