# Supplementary material for: Identification of OCTN2 variants and their association with phenotypes of Crohn’s disease in a Korean population
Source: Sci Rep. 2016 Mar 11;6:22887. doi: 10.1038/srep22887 (PMC4786794; doi:10.1038/srep22887)

Supplementary Information

For

Identification of *OCTN2* variants and their association with phenotypes of Crohn's disease in a Korean population

Hyo Jin Park[1], Eun Suk Jung[2,3], Kyoung Ae Kong[4], Eun-Mi Park[1], Jae Hee Cheon[3,*], Ji Ha Choi[1,*]

[1]Department of Pharmacology, Tissue Injury Defense Research Center, School of Medicine, Ewha Womans University, Seoul, 07985, Korea

[2]Department of Pharmacology, Brain Korea 21 PLUS Project for Medical Sciences, Severance Biomedical Science Institute, Yonsei University College of Medicine, Seoul, 03722, Korea

[3]Department of Internal Medicine and Institute of Gastroenterology, Yonsei University College of Medicine, Seoul, 03722, Korea

[4]Clinical Trial Center, Ewha Womans University Medical Center, Seoul, 07985, Korea

[*]corresponding authors: jihachoi@ewha.ac.kr or GENIUSHEE@yuhs.ac

Correspondence to:

Ji Ha Choi, M.D., Ph.D.

Department of Pharmacology, Tissue Injury Defense Research Center, School of Medicine, Ewha Womans University

1071 Anyangcheon-ro, Yangcheon-gu, Seoul, 07985, Korea

Tel: +82-2-2650-5746; Fax: +82-2-2653-8891; E-mail: jihachoi@ewha.ac.kr

Jae Hee Cheon, M.D., Ph.D.

Department of Internal Medicine and Institute of Gastroenterology, Yonsei University College of Medicine

50 Yonsei-ro, Seodaemun-gu, Seoul, 03722, Korea

Tel: +82-2-2228-1990; Fax: +82-2-393-6884; E-mail: GENIUSHEE@yuhs.ac

Supplementary Table S1. Frequencies of *OCTN2* genetic variations in promoter region from CHB/JPT population data

rs Number	Variant	Minor allele	Frequency	
			CHB	JPT
rs2631372	g.-2087G>C	C	0.325	0.399
rs2631371	g.-1974G>A	A	0.325	0.394
rs2631370	g.-1889T>C	T	0.422	0.274
rs186829555	g.-1679C>G	G	-	0.010
rs34786243	g.-945T>G	G	0.228	0.327
rs4646298	g.-446C>T	T	0.228	0.327
rs2631369	g.-399G>C	C	0.325	0.394
rs2631368	g.-368T>G	G	0.325	0.394
-	g.-299G>C	C	-	-
rs4646300	g.-234C>G	G	0.044	0.014

Data was obtained from 1000 Genomes Project (phase 3).
CHB (Han Chinese in Beijing, China), JPT (Japanese in Tokyo, Japan).

Supplementary Table S2. Frequencies of common *OCTN2* haplotypes in the promoter region from diverse populations

ID	g.-2087 G>C	g.-1974 G>A	g.-1889 T>C	g.-1679 C>G	g.-945 T>G	g.-446 C>T	g.-399 G>C	g.-368 T>G	g.-234 C>G	Frequency		
										ASW	CEU	CHB
H1	G	G	T	C	T	C	G	T	C	0.674	0.601	0.422
H2	**C**	**A**	**C**	C	T	C	**C**	**G**	C	0.152	0.273	0.282
H3	G	G	**C**	C	**G**	**T**	G	T	C	0.053	0.116	0.228
H4	G	G	**C**	C	T	**T**	**C**	**G**	C	0.076	-	-

The SNPs were marked in bold-faced letters and the minor alleles were marked in letters with underlines.
Data was obtained from 1000 Genomes Project (phase 3).
ASW (Americans of African ancestry in SW USA), CEU (Utah residents with Northern and Western European ancestry).

Supplementary Table S3. Frequencies of functional *OCTN2* genetic variations in patients and control groups

Variant		Patient, n (%)	Control, n (%)	*P* value
g.-1889T>C	+ / +	30 (15.5)	36 (12.8)	0.748
	+ / -	86 (44.6)	137 (48.8)	(0.398)
	- / -	77 (39.9)	108 (38.4)	
g.-945T>G	+ / +	111 (57.5)	151 (53.7)	0.277
	+ / -	71 (36.8)	118 (42.0)	(0.417)
	- / -	11 (5.7)	12 (4.3)	

The *P* values were obtained using recessive model (+/+ or +/- vs. -/-) or dominant model (+/+ vs. +/- or -/-). The values in parenthesis were from dominant model.

Supplementary Table S4. Results of the univariate Kaplan-Meier log-rank test analysis within patients

Parameter	Log-rank *P*
Surgery	0.015
Behavior	
Stricturing	0.310
Penetrating	0.357
Azathioprine or anti-TNF use	0.855

Supplementary Table S5. Oligonucleotides used in this study

Primes for *OCTN2* promoter cloning[a] (-2,490 to +128; 2,618 bp)	
Sense (KpnI site)	5’-CA**G GTA CC**A CTG GAC GGC ACC ATC TTA G-3’
Antisense (NheI site)	5’-CA**G CTA GC**A TCA GGA ACA CGG AGG ACA G-3’
Primers for *NF-E2* cloning[a]	
Sense (HindIII site)	5’-CG**A AGC TT**C TAG AGC CAT CTG GGC TGT C-3’
Antisense (XhoI site)	5’-CG**C TCG AG**A ATC CCA TCA GCA GTT CCA C-3’
Primers for *OCTN2* mutagenesis PCR[b]	
g.-2087G>C	5’-GAA GGT ATA ACC ATC CAT CTT CA**C** GAA GTA AGA ATA GTG AAT AGT AA-3’
g.-1974G>A	5’-TAT GCA AAT GTT TAG ATT TCC TT**A** TTA AAA ATC TGC TGG CCA GGT TA-3’
g.-1889T>C	5’-CTC ATA ATA TAG TGA CTA TTA AAT ATT CTT TAC **C**CA ACA CAT TAT ATT GTA AAA ATA TAC ACT TAG T-3’
g.-945T>G	5’-CTA TAT TTT TAT TTG CTA AAT CTG GCA ATT TTA TTA ATA **G**GG CTA CTA GAA AAG TTA ACA-3’
g.-446C>T	5’-CTA GAG GAG CGA GTT **T**GG ACT CGG ACC CC-3’
g.-399G>C	5’-GCC TTC CTA AGC CGA **C**CC CGG GCT-3’
g.-368T>G	5’-TCC CCA GCA GGC **G**TG GCT GGC AGA G-3’
Oligonucleotides for EMSA	
Reference (g.-1889T)[b]	5’-AAT ATT CTT TAC **T**CA ACA CAT TAT A-3’
Variant (g.-1889C)[b]	5’-AAT ATT CTT TAC **C**CA ACA CAT TAT A-3’
Consensus NF-E2[c]	5’-CCT CCA G**TG ACT CA**G CAC AGG TTC C-3’
Mutant consensus NF-E2[d]	5’-CCT CCA GTG **CTG TC**G CAC AGG TTC C-3’
Reference (g.-945T)[b]	5’-ATT TTA TTA ATA **T**GG CTA CTA GAA A-3’
Variant (g.-945G)[b]	5’-ATT TTA TTA ATA **G**GG CTA CTA GAA A-3’
Consensus YY1[c]	5’-CGC TTC CCG G**CC ATC T**TG GCG GCT GGT-3’

[a]The restriction endonuclease sites were marked by bold-faced letters with underlines.
[b]The SNP sites were marked by bold-faced letters with underlines.
[c]The consensus sequences of transcription factors were marked by bold-faced letters with underlines[48, 58].
[d]The changes in consensus sequences were marked by bold-faced letters with underlines.

Supplementary Figure S1. LD structures of *OCTN2* promoter variants in three ethnic populations. Data was obtained from the 1000 Genomes Project (phase 3, https://www.ncbi.nlm.nih.gov/variation/tools/1000genomes/).

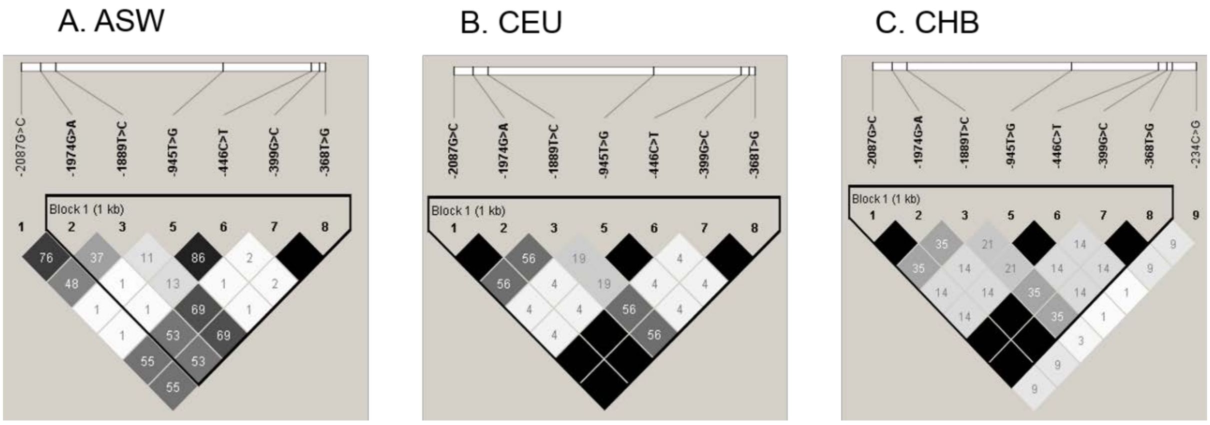

Supplement: Supplementary Information [file srep22887-s1.pdf]
